# Investigation of FBG Accelerometers and Load Sensors for Vibration Analysis and Slip Detection in Robotic Manipulators

**DOI:** 10.3390/s26196176

**Published:** 2026-09-29

**Authors:** Juraj Kováč, Viktor Hrúz, Ľuboš Chovanec, Izabela Trepáčová, František Duchoň, Peter Hubinský, Zuzana Kovaríková, Roman Mykhailyshyn

**Affiliations:** 1 Institute of Robotics and Cybernetics, Faculty of Electrical Engineering and Information Technology, Slovak University of Technology in Bratislava, Ilkovičova 3, 812 19 Bratislava, Slovakia; viktor.hruz@stuba.sk (V.H.); lubos.chovanec@stuba.sk (Ľ.C.); izabela.trepacova@stuba.sk (I.T.); frantisek.duchon@stuba.sk (F.D.); peter.hubinsky@stuba.sk (P.H.); 2 VÚEZ, a.s., Hviezdoslavova 35, 934 39 Levice, Slovakia; kovarikova@vuez.sk; 3 National Institute of Advanced Industrial Science and Technology (AIST), Tokyo Waterfront, 2-3-26 Aomi, Koto-ku, Tokyo 135-0064, Japan; roman.mykhailyshyn@aist.go.jp

**Keywords:** Fiber Bragg Grating (FBG), fiber optic, vibration analysis, slip detection, robotic manipulator, robotic gripper, predictive maintenance

## Abstract

This paper presents an investigation of the viability of Fiber Bragg Grating (FBG) accelerometers and load sensors for two diagnostic tasks in robotics: vibration analysis of industrial manipulators and slip detection in robotic grippers. The study synthesizes experimental results from two independent experiments, focusing on the use of optical sensors in place of traditional electromechanical devices such as MEMS accelerometers and resistive strain gauges. The experiments were performed on an ABB industrial robot with a parallel robotic gripper equipped with embedded optical fibers. Under the conditions tested, the optical chains provided good sensitivity and detected dynamic events such as slip onset and contact or movement-induced vibrations. Compared with the conventional chains recorded alongside them, the fiber-optic accelerometers delivered cleaner vibration spectra (SNR 36.7 dB against 13.7 dB), while the FBG load sensors measured gripping forces with a lower noise floor and less mains-band interference, although the force lost before a slip alarm favored the FBG chain only in the experiment carried out with brass. Obtained with a single specimen of each sensor type and a limited set of grasped objects, these findings indicate that optical sensing is a viable alternative for robotic diagnostics, but it comes at the cost of higher price and complexity.

## 1. Introduction

Modern robotic systems increasingly operate in environments where the precision of movement, stability of manipulation and reliability of diagnostic processes play a crucial role in overall performance. Robots performing assembly, inspection or handling tasks must detect not only their own mechanical state but also the state of the objects they manipulate. Among others, two sensing capabilities are fairly interesting in this context: vibration analysis, which evaluates internal mechanical behavior through vibration patterns [1,2], and slip detection, which ensures stable manipulation by identifying when an object begins to move unintentionally within the gripper [3,4].

Traditional sensing technologies used for these tasks, such as piezoelectric accelerometers, MEMS inertial modules or resistive strain gauges, remain common in industrial environments due to their low cost and relatively straightforward integration. However, these sensors tend to be sensitive to electromagnetic interference, temperature variations, moisture and corrosive or otherwise extreme environments. For this reason, electromechanical sensors can struggle to provide reliable measurements in dynamic or harsh industrial settings [5].

In recent years, fiber-optic sensors, and particularly Fiber Bragg Grating (FBG) technology, have emerged as a promising alternative. Fiber Bragg Grating sensors operate on the principle of periodic modulation of the refractive index within the core of an optical fiber. This structure forms a wavelength-selective mirror that reflects a narrow spectral component, i.e., the Bragg wavelength. Because the parameters of the Bragg grating vary with strain and temperature, the Bragg wavelength shifts in response to mechanical deformation through changes in grating pitch and the stress-optic effect, and in response to temperature fluctuations via thermal expansion and the thermo-optic effect [2,5,6,7,8]. These properties enable FBG sensors to function as precise sensors, bringing many advantages compared to traditional electromechanical sensors.

FBG sensors are fairly common and are being actively explored as alternatives to traditional sensors in many other fields thanks to their versatility and numerous other advantages, like high sensitivity, light weight, small size, biocompatibility, corrosion resistance, immunity to electromagnetic interference (EMI), electrical insulation, passive operation, the ability to be embedded into materials or structures, their intrinsic safety, especially in flammable environments and environments with a risk of explosion, and the possibility for remote operation [5,7,8,9,10,11,12,13]. These sensors are highly explored in construction and structural health monitoring of bridges, roads, dams, tunnels, smart buildings, railways, pipelines and other structures [5,7,8,9]. Similarly, they are used in the aerospace industry for monitoring the structural health of flexible aerospace parts like wings [5,10]. Thanks to their resilience, safety, sensitivity and remote operation possibilities, they are used in the oil and gas industry for tasks like structural health monitoring, exploration by seismic surveying and temperature and pressure monitoring in oil wells [11]. They have become quite attractive for medical applications due to their unique properties, such as their small size, biocompatibility, immunity to electromagnetic interferences, high sensitivity and multiplexing capability [12]. Thanks to the versatility of Fiber Bragg Grating, they can be used for the environmental sensing of many different environmental properties, like temperature, pressure, gas detection, water quality measurements and many more [5]. However, their usage in industrial robotics has still not been explored very deeply. This may be due to some of their disadvantages, like their higher cost, increased complexity, more complicated installation and the potential mechanical fragility of the fiber-optic lines. However, many of these issues are being addressed and have been significantly improved in recent years. The goal of this article is to investigate the viability of these sensors in the fields of vibration analysis of robotic manipulators and slip detection for robotic grippers.

Published work on FBG sensing in robotics is divided between two lines that rarely meet. Accelerometer studies concentrate on transducer design and characterize the resulting device on a shaker table or, where an external reference is used at all, against geophones in seismic field trials [14,15], and the application domains surveyed for FBG vibration sensing, like geohazards, oil and gas, civil infrastructure and rotating machinery, do not include industrial manipulators [16]. FBG tactile and slip sensors, conversely, are demonstrated on robotic fingertips and grippers but are evaluated on their own terms, without a quantitative baseline from the electrical sensing chain they are proposed to replace [17,18,19]. Where a contrast with resistive strain gauges is drawn, it is drawn in terms of the sensor count, wiring complexity and susceptibility to interference rather than from measurements acquired under identical conditions [20]. The present work addresses this gap by operating commercial FBG accelerometer and load sensors on an ABB IRB 4600 manipulator and a parallel gripper alongside a MEMS accelerometer and a resistive strain gauge chain, interrogating both modalities on the same instrument and comparing them on shared metrics across repeated runs. Doing so allows the properties usually asserted for FBG sensing in robotics, like sensitivity, low-frequency fidelity and immunity to electromagnetic interference, to be quantified rather than inferred. The interference advantage is resolved spectrally, with a mean of 86.4% ± 3.1% for the resistive chain’s idle noise falling in the mains band against roughly 11.3% ± 1.3% for the FBG chain, and it is then translated into an operational consequence through the force lost before a slip alarm, a cross-chain metric that requires no clock alignment.

## 2. Materials and Methods

The experimental investigation was divided into two complementary parts: vibration analysis of the manipulator and slip detection in the gripper. The measurement chains used in both parts are summarized in Table 1.

### 2.1. Vibration Analysis

**Figure 1:**
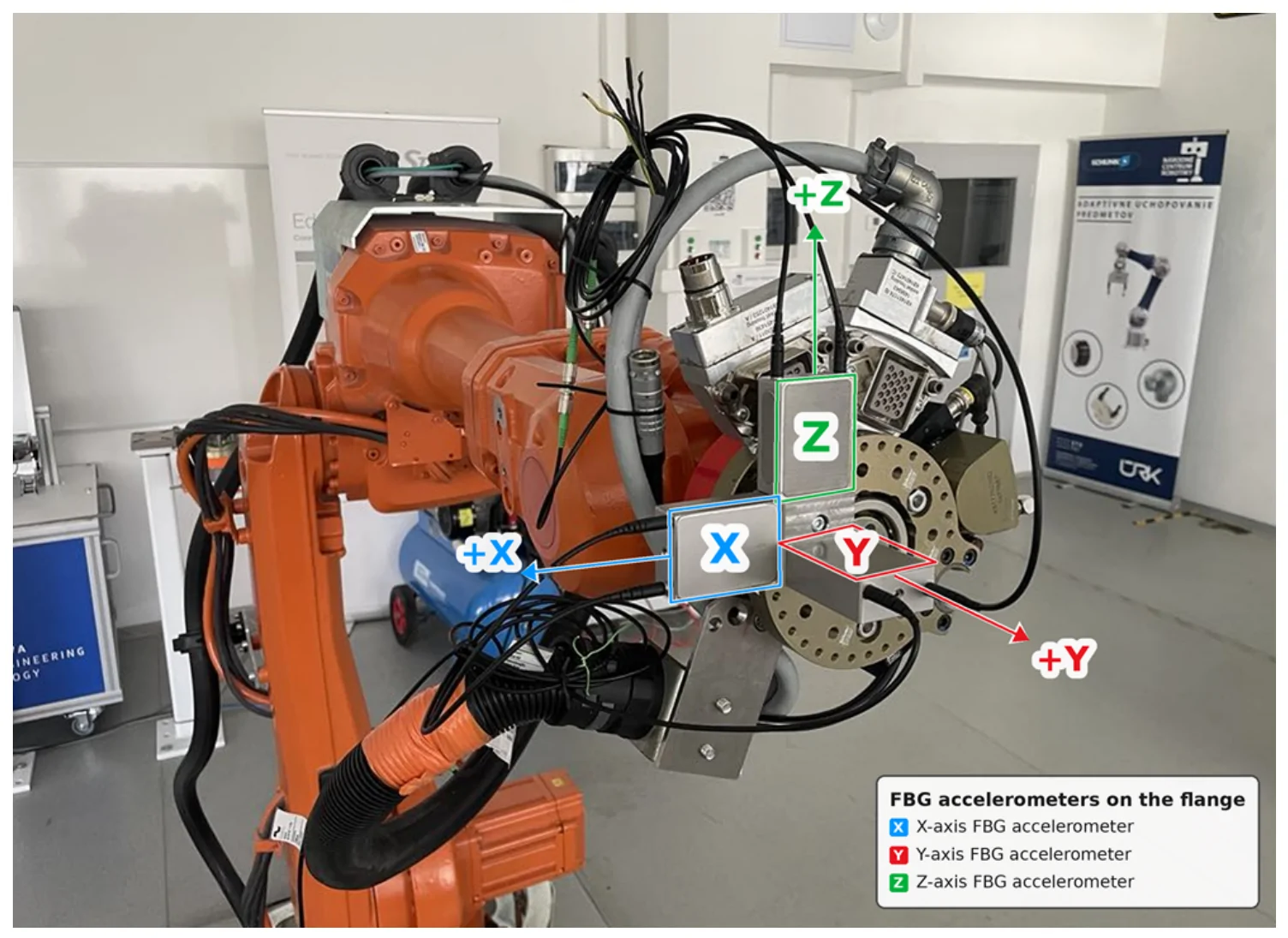
ABB IRB 4600 robot equipped with a set of three SAA-02 fiber-optic accelerometers. Colored boxes mark the X, Y and Z axis accelerometers, and arrows show the positive direction of each measurement axis.

The first part focused on the vibration analysis of an ABB IRB 4600 robot. A set of three SAA-02 single-axis fiber-optic accelerometers was mounted on the flange of the robot (Figure 1), oriented so that each measured a different axis. These were connected to an SCN-80 S-line Scan 800 interrogator through an S-line Switch1x16D optical switch. This way, the measurement system measured acceleration for all three axes (X, Y, Z); however, the analysis was carried out on the axis that the robot flange moved in during an experiment. Additionally, a MEMS-based MPU-6050 accelerometer along with an Arduino nano was used for comparison. Both accelerometers were configured to sample at a frequency of 800 Hz, which enabled spectral analysis up to 400 Hz according to the Nyquist–Shannon sampling theorem [21]. This analysis was carried out using the Fast Fourier Transform (FFT) algorithm [22,23]. Specifically, mean-removed time segments were transformed by the Fast Fourier Transform (FFT) with a Hann window and coherent-gain correction (ENBW =1.5 bins) and without zero padding, yielding single-sided amplitude spectra with a bin width Δf=1/T set by the segment duration *T*. In the accelerometer comparison, the FBG and MEMS segments were additionally matched in length within each run so that both chains shared a common Δf.

Multiple experiments were conducted with this setup, with the robot flange moving along a rectangular trajectory. Each conducted experiment was repeated 10 times. Since the results of the experiments did not meaningfully differ between runs, the first run of each experiment was chosen as representative and is shown in the figures. All 10 runs are, however, evaluated in Table 2. Robot motion is inherently non-stationary, so each window was placed over a nominally steady portion of the trajectory, and the reported spectra and RMS values are to be read as window averages rather than instantaneous quantities. Repeating the analysis 10 times with independently re-selected windows quantified the sensitivity of the metrics to that placement, which is reflected in the standard deviations of Table 2.

The first experiment was focused on a comparison of a more traditional MEMS accelerometer with the FBG accelerometer. In this experiment, the robot completed the trajectory with both sensors recording simultaneously. The data from the X axis accelerometers were analyzed using FFT, to compare the quality of the measured signals from the two accelerometers.

In the second experiment, the effect of speed of the robot on vibrations was tested. The robot equipped with the fiber-optic accelerometers completed the trajectory from the previous experiment with different speeds, specifically, 0.25 m/s, 0.5 m/s and 1 m/s. The X and Z axis data were then analyzed.

In the third experiment, the effect of a weighted payload on vibrations was investigated. Again, the robot completed the same trajectory, first without any payload and subsequently with a 20 kg payload. Similarly to in the previous experiments, the X axis data were analyzed using FFT.

The fourth experiment tested the ability to detect a mounting fault, specifically, loose mounting of a payload. The robot completed the trajectory from the previous experiment with a payload of 20 kg mounted on the flange. In the first set of experiments, the payload was mounted securely, and in the other set of experiments, the screws holding the payload were loosened so it could move slightly. Again, the X axis vibration data were analyzed.

The last experiment focused on the ability to detect a collision of the payload with an object. First, the robot completed the trajectory without any obstacles in its way. Afterwards, an object with a mass of 20 kg was placed in the path of the robot and the trajectory was repeated. The robot flange was moving along the Y axis during the collision, so the Y axis data were analyzed.

### 2.2. Slip Detection

**Figure 2:**
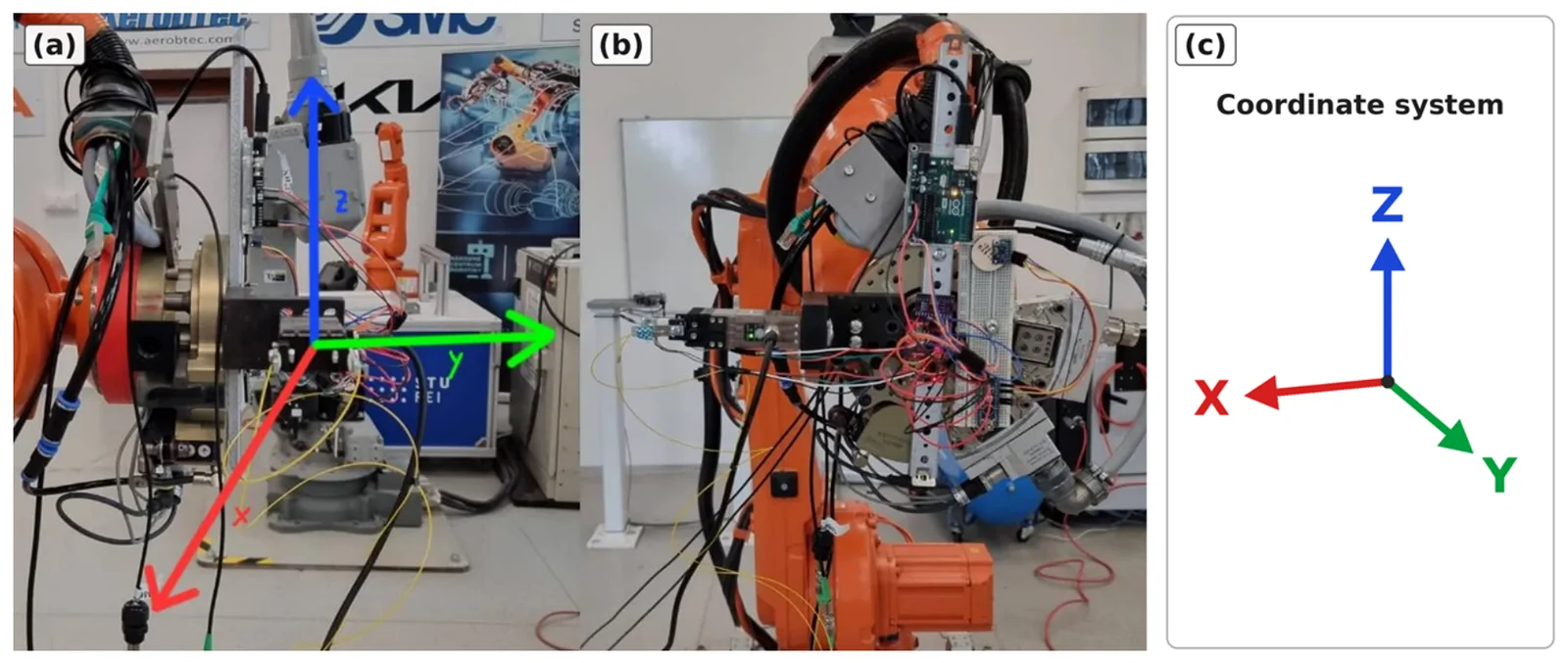
ABB IRB 4600 robot equipped with a gripper and load sensors: (**a**) side view; (**b**) front view; (**c**) robot tool coordinate system used in (**a**), with the X (red), Y (green) and Z (blue) axes.

The second part of the investigation examined slip detection of an object in a parallel gripper using load cells. Two SWA-00 FBG load sensors were embedded into milled grooves in the gripper fingers (Figure 2) so that mechanical strain induced by gripping forces produced corresponding wavelength shifts in the optical fibers. An SCN-80 S-line Scan 800 optical interrogator captured these shifts at a sampling frequency of 800 Hz, enabling monitoring of force variations during manipulation. For comparison, resistive strain gauges were bonded to the same gripper fingers and connected through an NAU7802 load cell amplifier, which was connected to an Arduino Uno and sampled with its maximum 320 Hz frequency. The experiments were conducted with three payload materials: wood, rubber and brass. Each of them was tested by moving them along a rectangular trajectory to induce slipping. The performance of the FBG sensors and strain gauges was evaluated using stability of force measurement, clarity of slip signatures and resistance to noise. Multiple experiments were conducted, during which the ABB IRB 4600 robot moved the effector along a square trajectory with a maximum speed of 1 m/s. Experiments were conducted at a stable temperature of 23 ± 0.5 °C. Every experiment was repeated five times. The first run of each set was chosen to be the representative experiment shown in the figures, since the results did not meaningfully deviate between runs; however, all five runs are evaluated in Table 3. The grip force was set to 35 N, which was the lowest force setting allowed by the gripper. This allowed the gripper to hold the object just enough for the object to be able to slightly slip.

In the first experiment, a wooden cube with a size of 3 cm and a mass of 16 g was gripped by the robot with a force of 35 N near the center of mass of the cube so we could observe the behavior of the system when no slipping occurred. Data from both the resistive and fiber-optic load sensors were gathered and analyzed. The second experiment was focused on slip detection of a wooden cube payload during constant-speed movement. The same wooden cube was gripped, this time farther from the center of mass, to induce slipping and rotation of the payload. The robot then completed the trajectory with a speed of 1 m/s two times. The third experiment was very similar to the second experiment, but rather than using a wooden payload, rubber was used, specifically, a rubber cube with a mass of 48 g. Lastly, the same experiment was repeated again, but this time with a brass object with a mass of 62 g.

Neither optical chain used anti-aliasing filtering. The MEMS reference ran with its digital low-pass at 260 Hz, the resistive bridge was digitized at 320 SPS with the NAU7802 mains notch inactive at that rate and force signals were smoothed with a centered 50-sample moving average before differentiation.

## 3. Results

### 3.1. Vibration Analysis

#### 3.1.1. Comparison of Sensors

**Figure 3:**
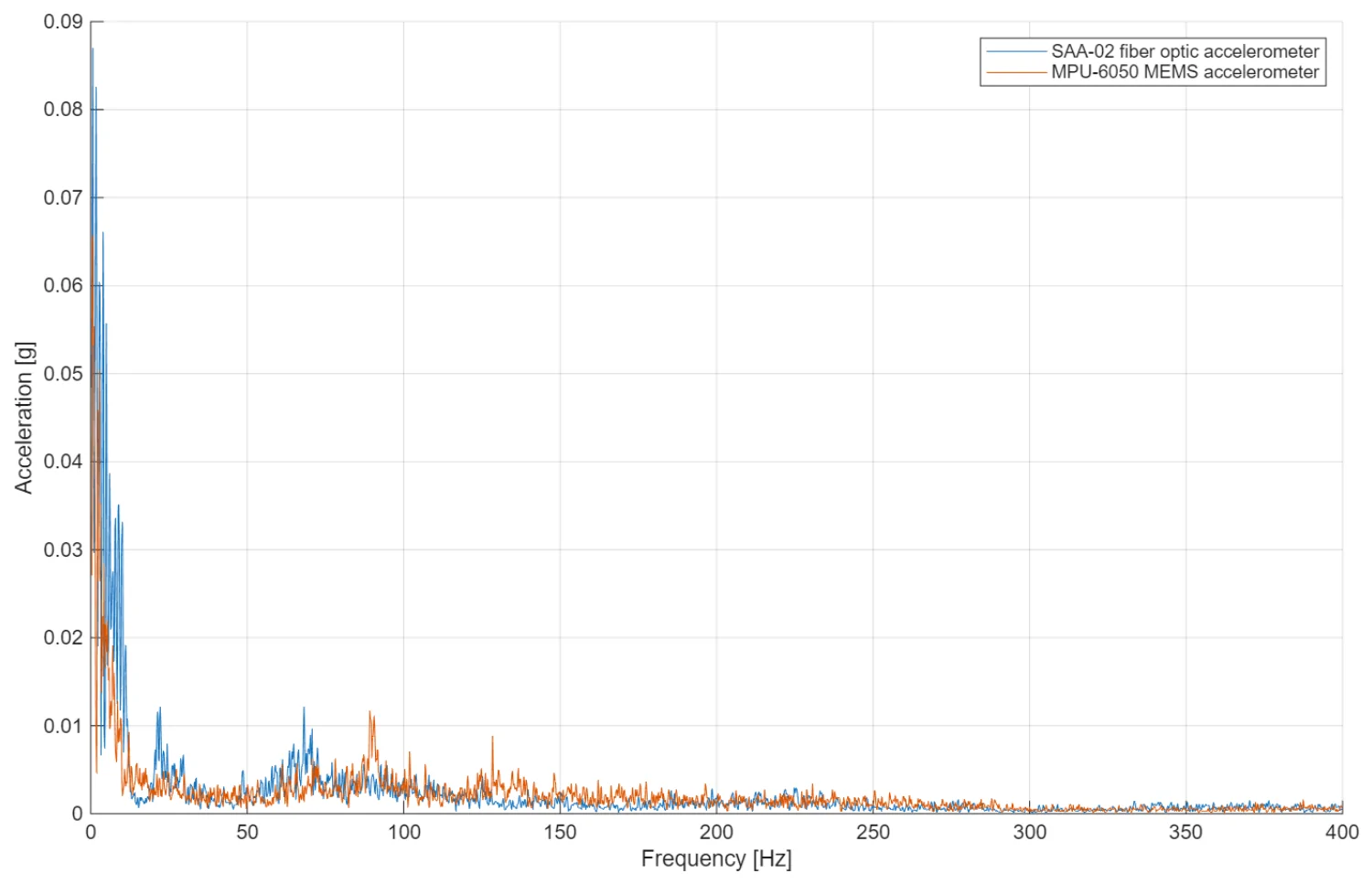
Frequency spectrum of robot vibration from the SAA-02 FBG accelerometer and the MPU-6050 MEMS accelerometer.

There was a clear difference between the two accelerometers, as shown in Figure 3. The MEMS accelerometer generated substantially noisier data, which complicated the analysis of the measured data. On the other hand, the SAA-02 fiber-optic accelerometer measured cleaner vibration data with a lower noise floor and a higher signal-to-noise ratio, which can also be seen in Table 2. The optical sensor also demonstrated higher signal amplitudes, especially in the 0 Hz–20 Hz range, compared to the MPU-6050. The most dominant frequency components were in the frequency range under 150 Hz.

#### 3.1.2. The Effects of Speed on Vibrations

**Figure 4:**
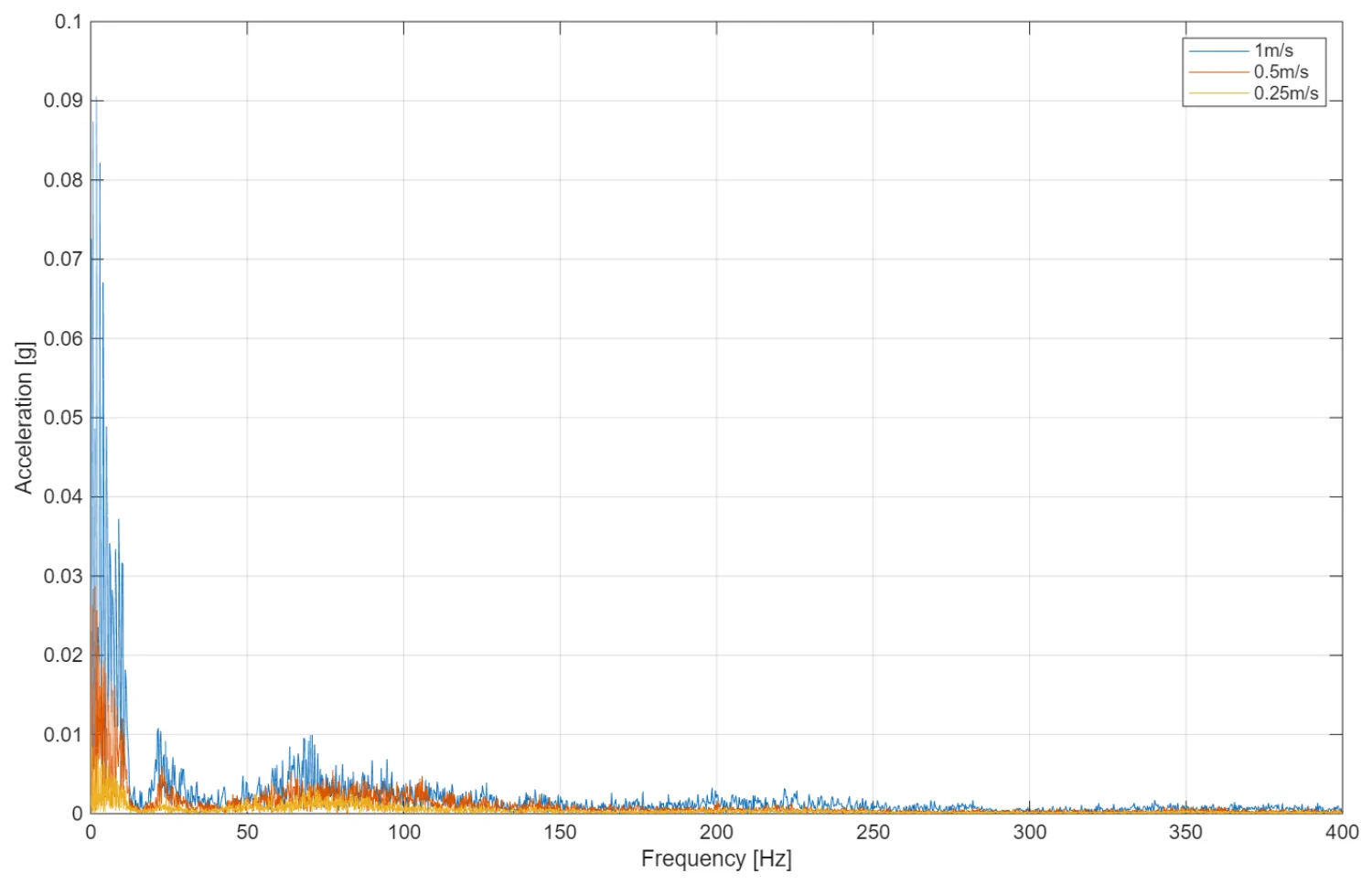
Frequency spectrum of robot vibration with different speeds, measured by the SAA-02 FBG accelerometer—X axis.

**Figure 5:**
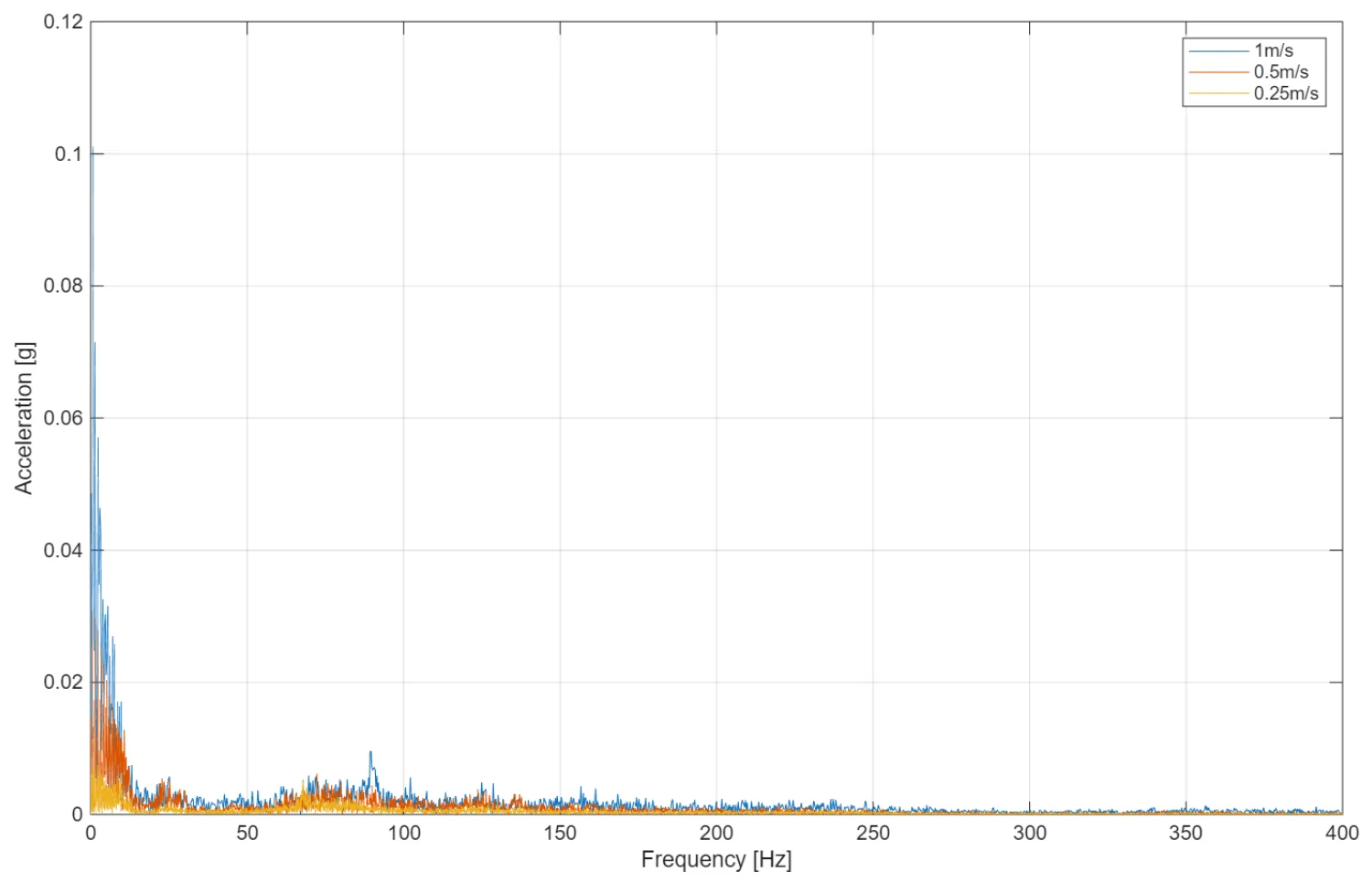
Frequency spectrum of robot vibration with different speeds, measured by the SAA-02 FBG accelerometer—Z axis.

With increasing speed, an increase in the amplitude of the vibrations was measured. These vibrations were more prominent on the X axis (Figure 4) compared to the Z axis (Figure 5). They were substantially higher during the experiment with a speed of 1 m/s, while, during the experiment with the lowest speed, the vibrations were greatly dampened.

#### 3.1.3. The Effects of a Weighted Payload on Vibrations

**Figure 6:**
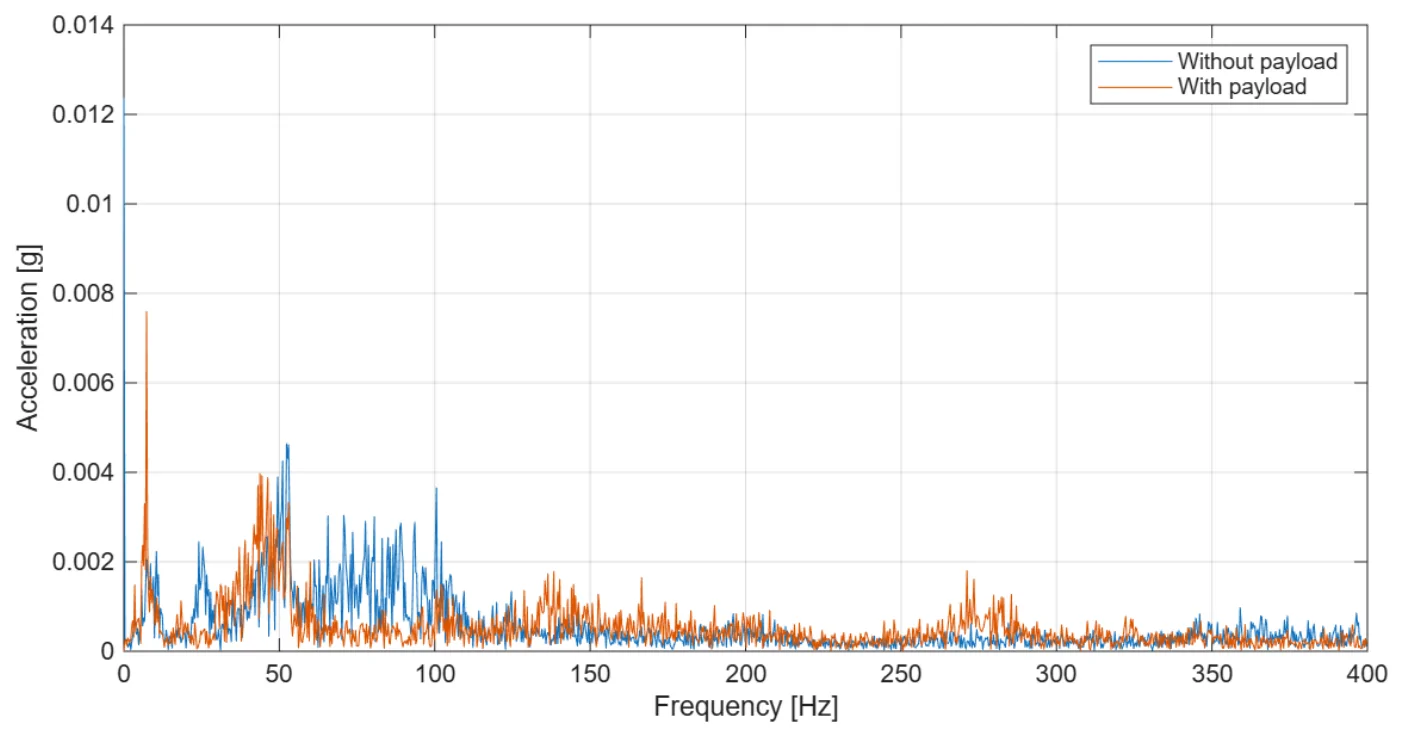
Frequency spectrum of robot vibration with and without payload, measured by the SAA-02 FBG accelerometer—X axis.

When comparing data measured with and without a payload, a clear difference in the vibrations of the robot was found (see Figure 6). The weighted payload caused a noticeable decrease in amplitudes in the frequency range of 60 Hz to 100 Hz. On the other hand, an increase in the amplitudes in the frequency range of 130 Hz to 300 Hz was observed with the payload equipped. These frequencies of vibrations were considerably dampened or not present during the experiments without a payload.

#### 3.1.4. Improper Payload Mounting Detection

**Figure 7:**
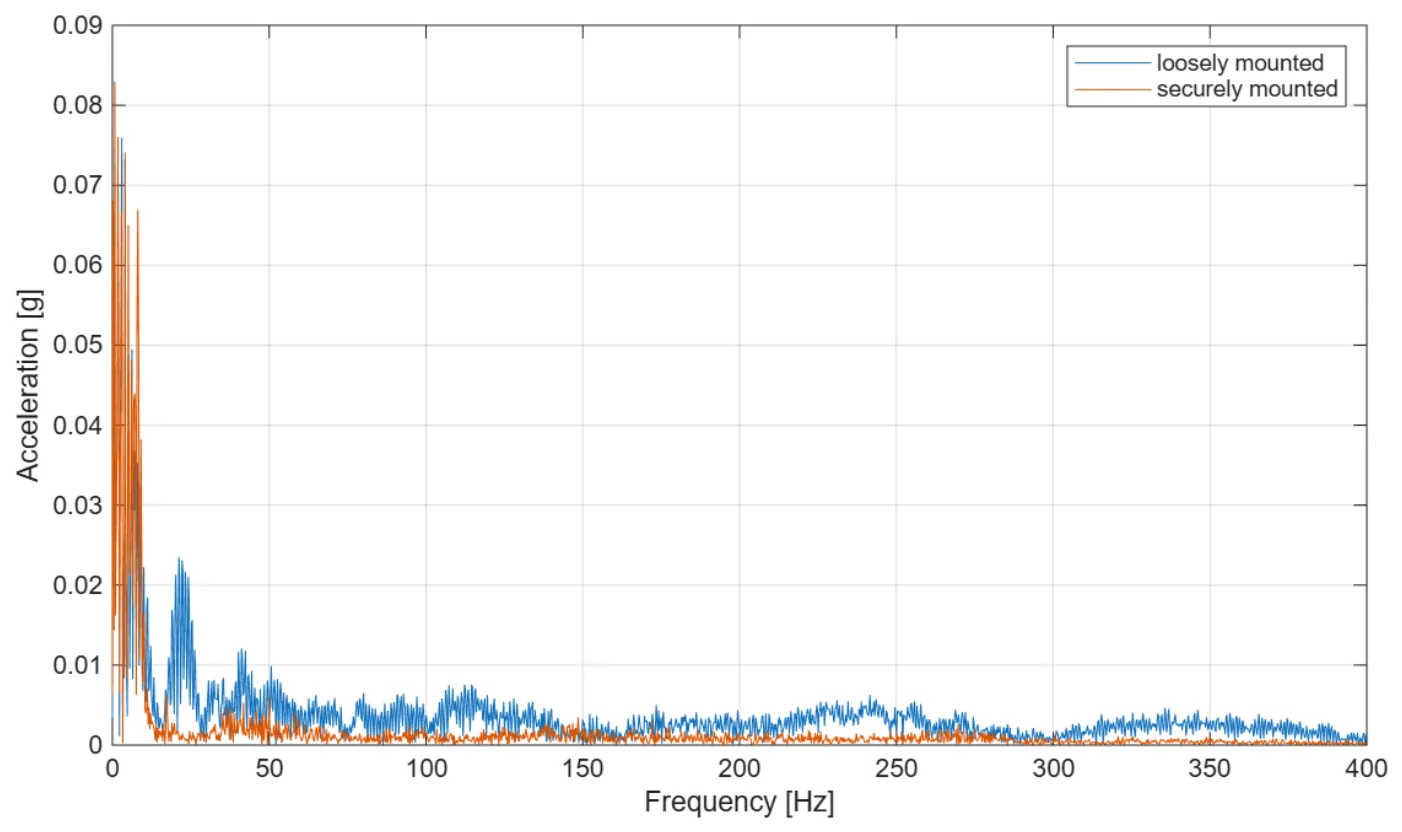
Frequency spectrum of robot vibration with securely and loosely mounted payloads, measured by the SAA-02 FBG accelerometer—X axis.

The experiment showed a noticeable difference between the vibration characteristics of a securely mounted payload and those of a loosely mounted payload, visible in Figure 7. With a securely mounted payload, most of the vibration energy was focused in the frequency range of 0 Hz to 10 Hz. In contrast, the experiments with the loosely mounted payload showed significant frequency components in the range of 20 Hz to 300 Hz which were not present with the securely mounted payload.

#### 3.1.5. Collision Detection

**Figure 8:**
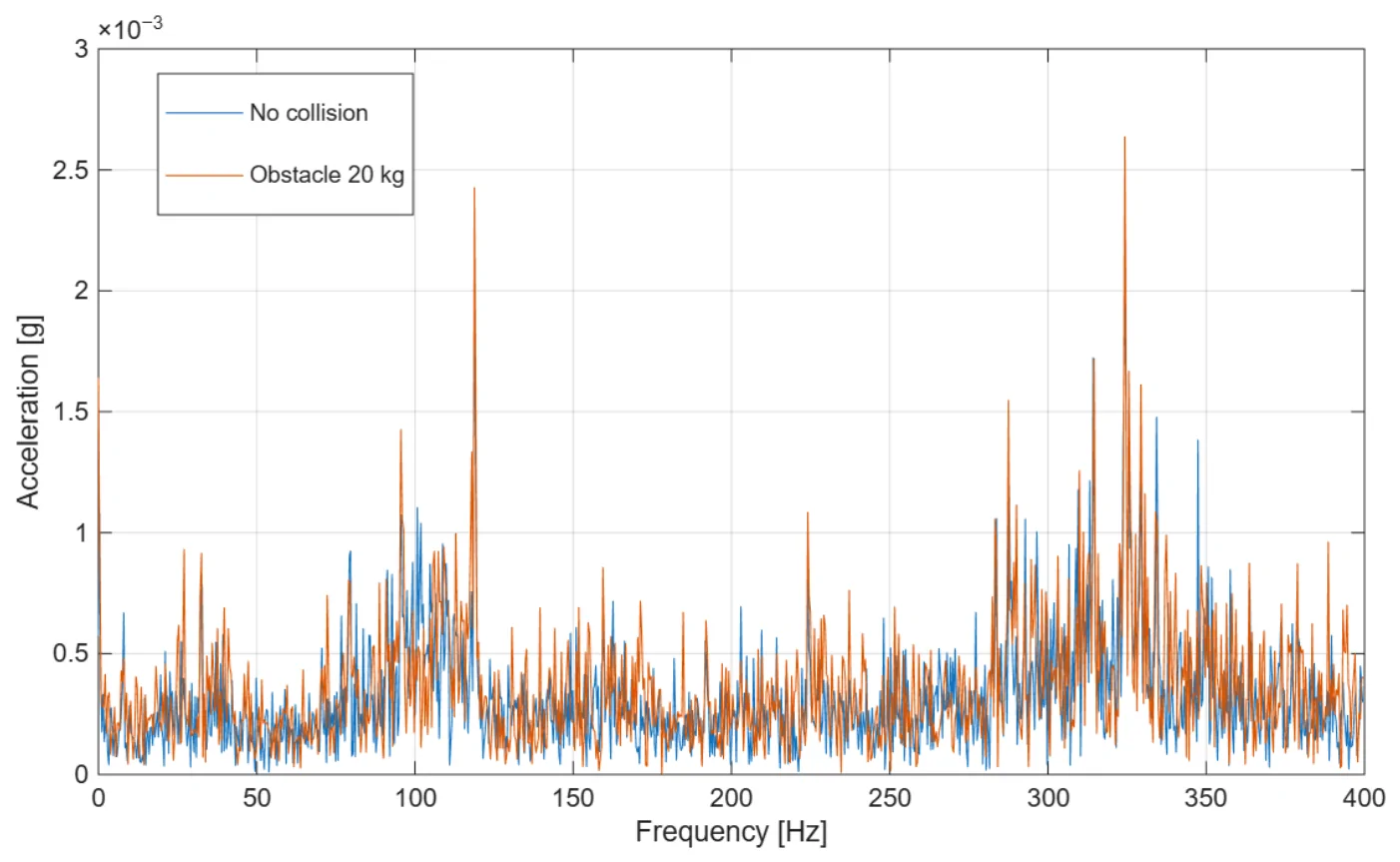
Frequency spectrum of robot vibration with and without collision, measured by the SAA-02 FBG accelerometer—Y axis.

The frequency characteristics of the vibrations were again different for the experiments with and without an obstacle in the robot path (Figure 8). New frequency components were observed, mostly near 120 Hz, 225 Hz, 290 Hz and 325 Hz, which were not present in the experiments without the obstacle. The amplitudes of vibrations also increased in the experiment with the obstacle.

### 3.2. Slip Detection

#### 3.2.1. Initial Experiment

**Figure 9:**
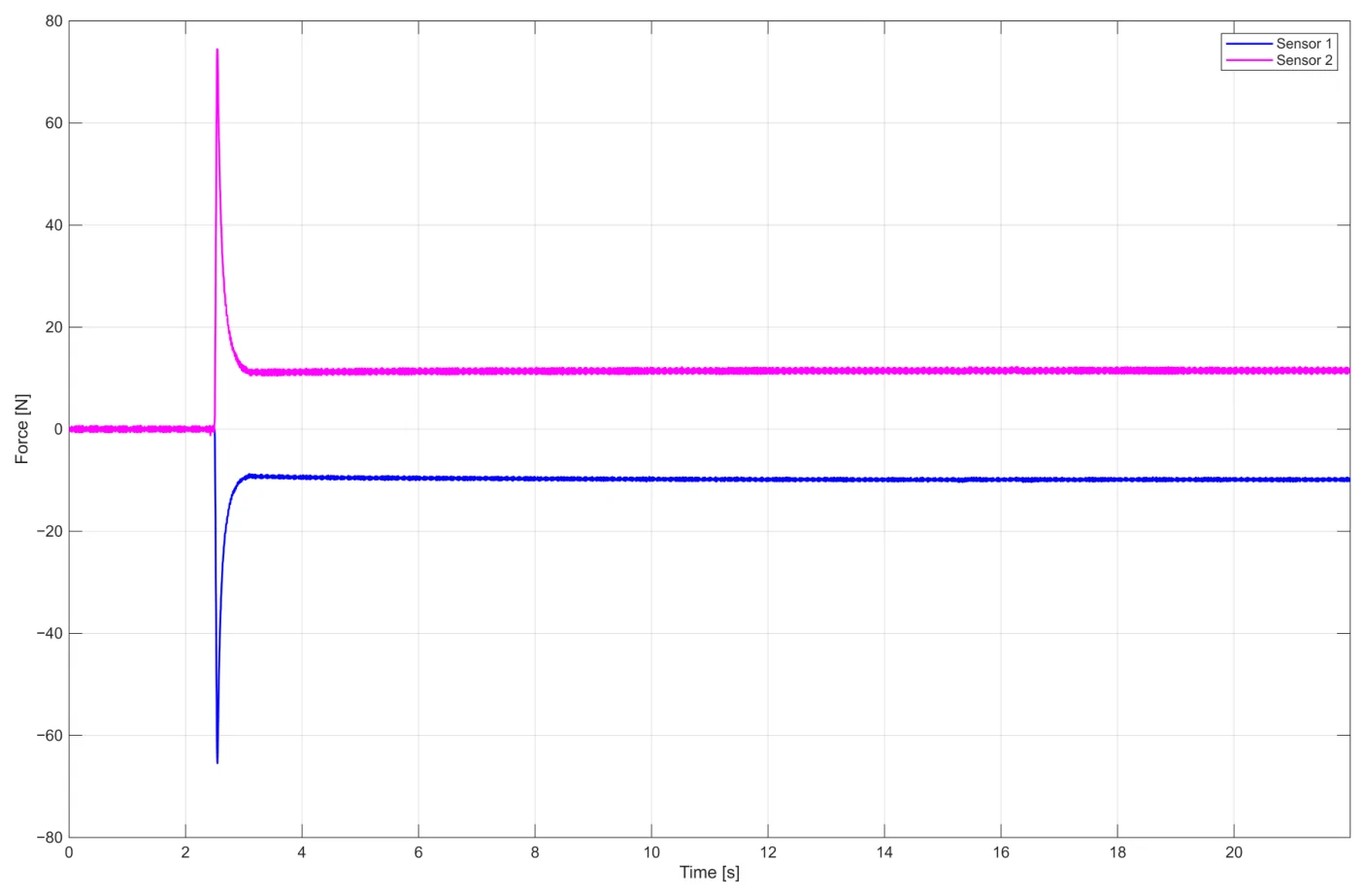
Forces measured by resistive load cells during the initial experiment (Finger 1 and Finger 2).

**Figure 10:**
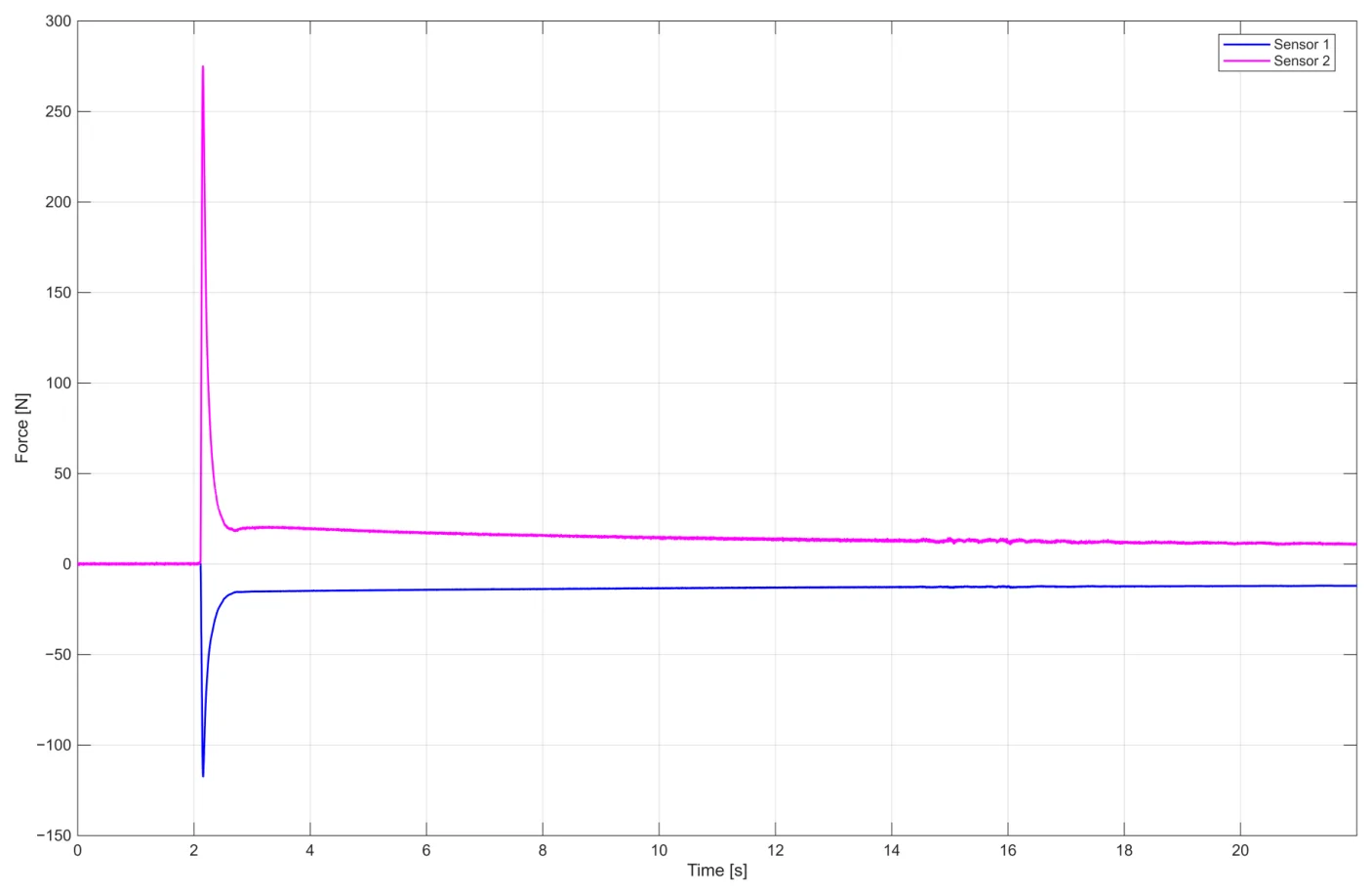
Forces measured by FBG load cells during the initial experiment (Finger 1 and Finger 2).

Since the object was securely gripped near the center of mass, no slipping occurred during this experiment. A large contact impulse of force, which occurred when the gripper made contact with the gripped object, was detected with both sensors. The hold force of the gripper then equalized close to the configured force of 35 N; however, the force measured by the sensors equalized at lower values (see Figure 9 and Figure 10). This behavior was observed in every experiment using the robotic gripper. Small fluctuations of force were present from 15 s to 17 s in measurements from both sensors; however, they were more visible in the data from the fiber-optic load sensor thanks to its cleaner signal.

#### 3.2.2. Wooden Object

**Figure 11:**
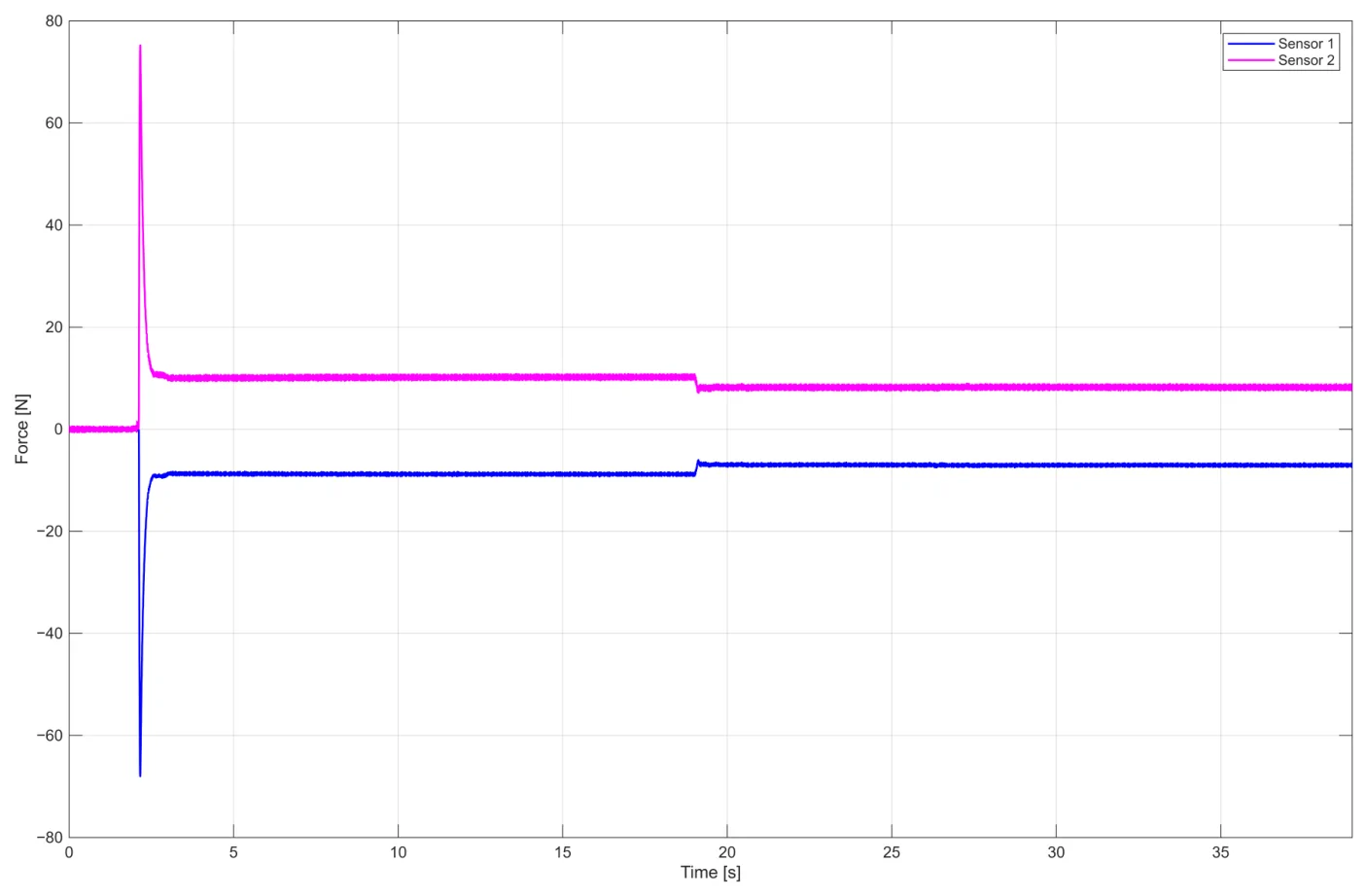
Forces measured by resistive load cells during the slip experiment with the wooden cube (Finger 1 and Finger 2).

**Figure 12:**
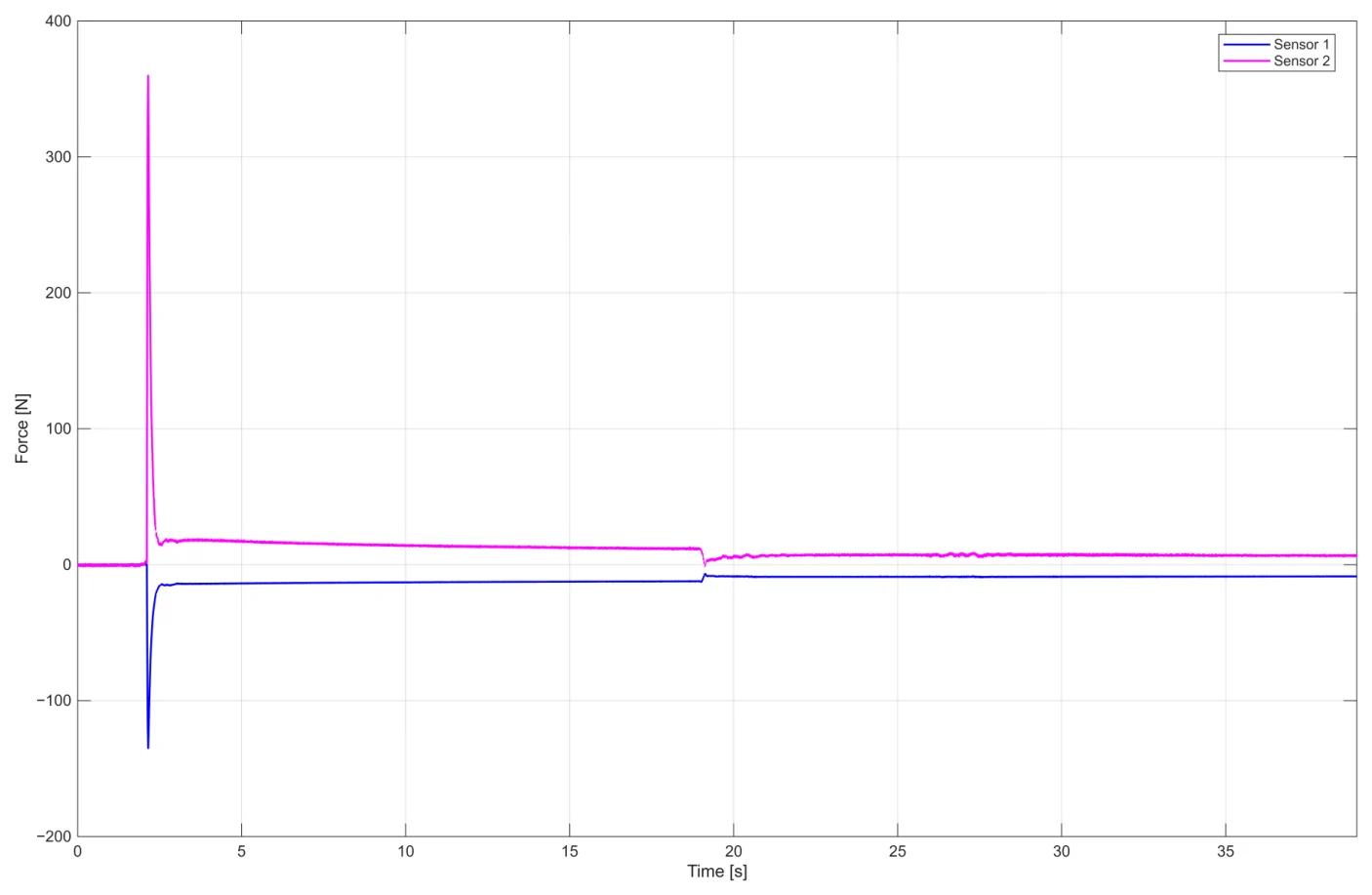
Forces measured by FBG load cells during the experiment with the wooden cube with slipping (Finger 1 and Finger 2).

During this experiment, slipping of the wooden cube occurred in the first cycle of the trajectory. Both load cells detected a sudden decrease in force at around 19 s, after which the force stabilized again, which is visible in Figure 11 and Figure 12. Right after the sudden decrease in force, small oscillations were measured as the force stabilized. These oscillations were very hard to spot in the data from the resistive load cell, but were considerably more visible in the data measured by the FBG load cell. Additional oscillations were observed in the data from the fiber-optic sensor in the second cycle of the trajectory (between 25 s and 30 s), where no visible slipping occurred. These oscillations were not visible in the resistive load cell data.

#### 3.2.3. Rubber Object

**Figure 13:**
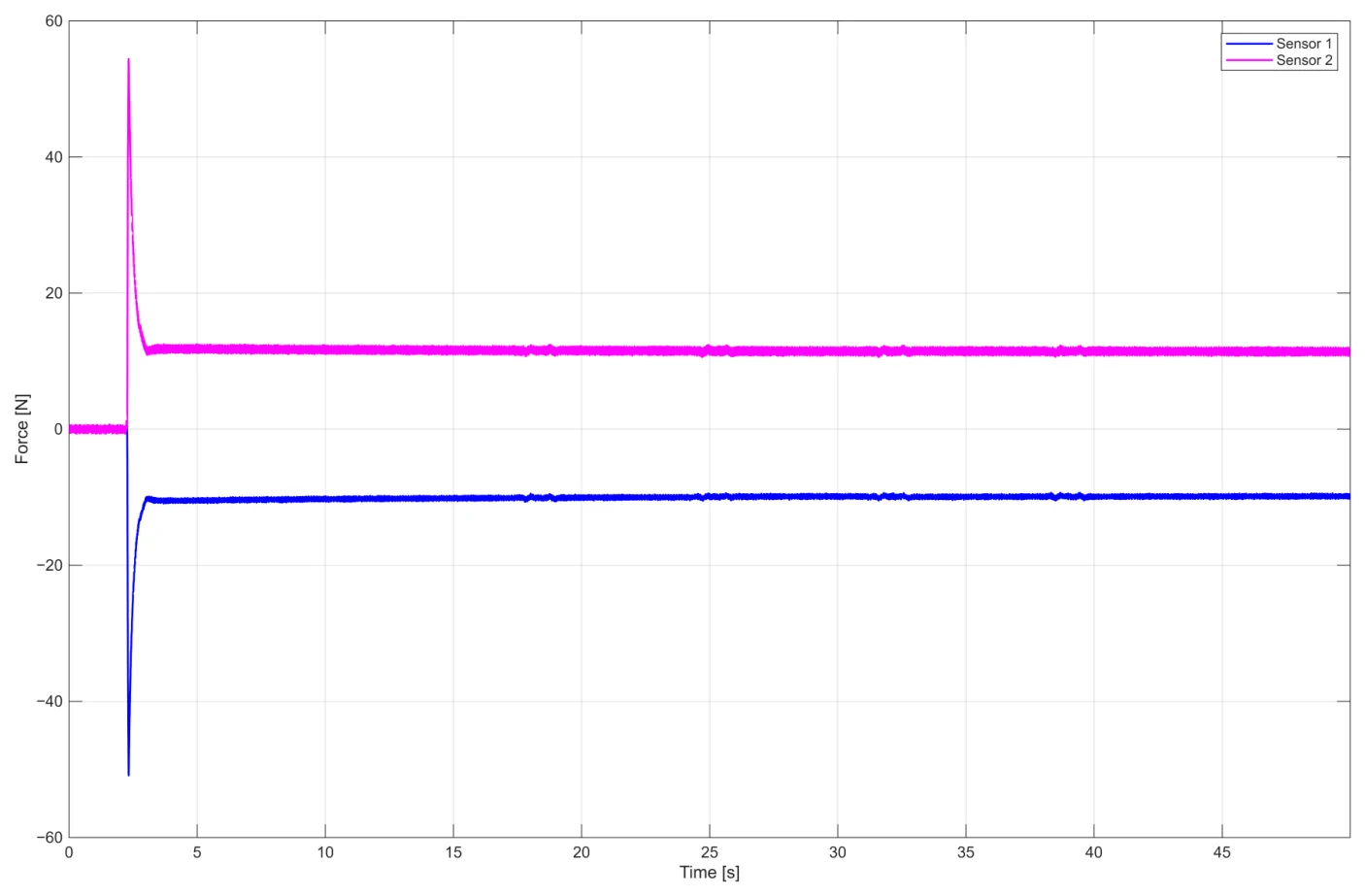
Forces measured by resistive load cells during the experiment with the rubber cube (Finger 1 and Finger 2).

**Figure 14:**
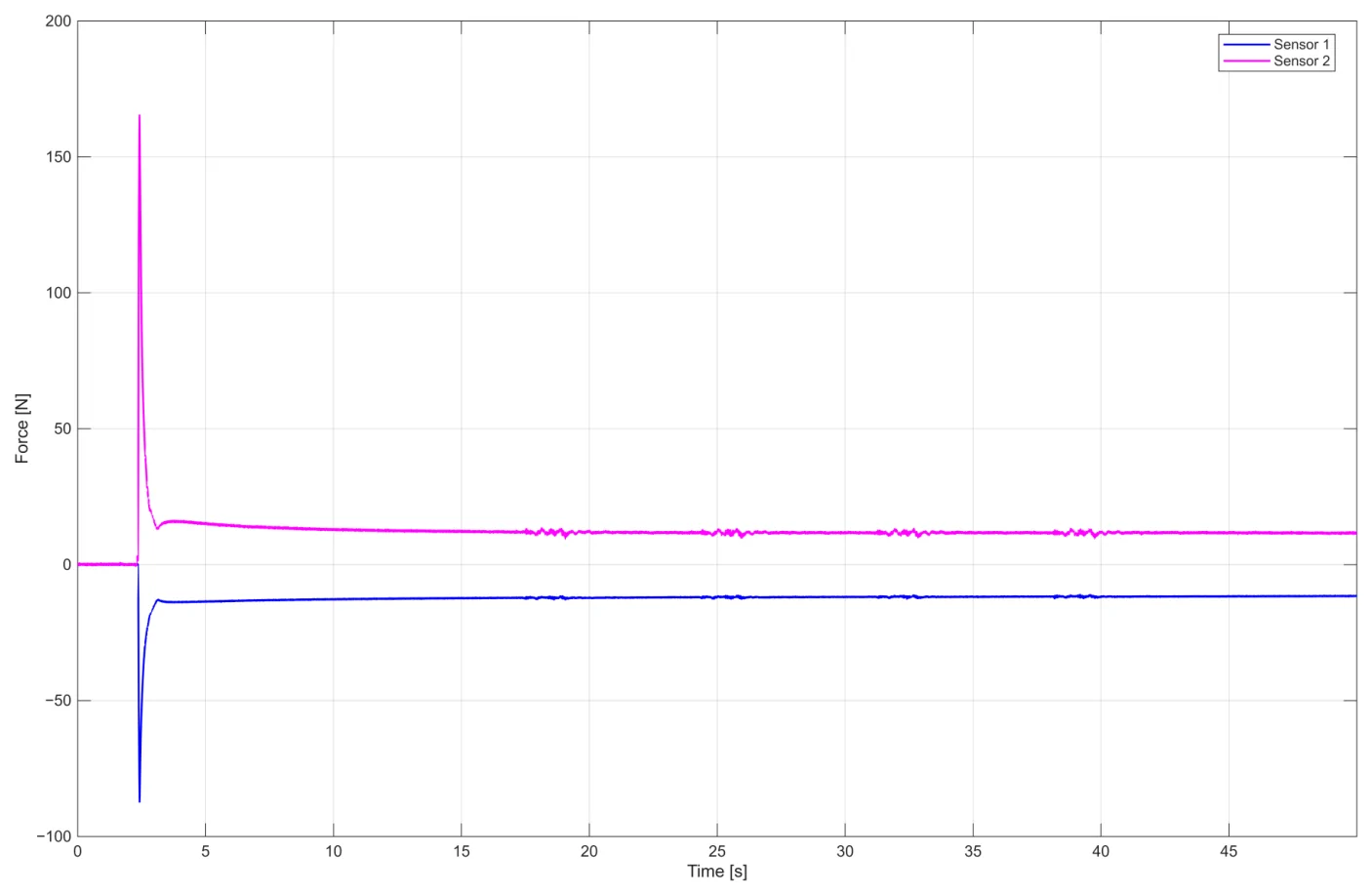
Forces measured by FBG load cells during the experiment with the rubber cube (Finger 1 and Finger 2).

The measured forces were quite stable during this experiment, other than, of course, the impulse of force at the moment of contact with the object (see Figure 13 and Figure 14). There were no other significant changes in the grip force, since no slipping occurred during this experiment. Slight oscillations in the grip force at around 17 s, 24 s, 32 s and 37 s were still detectable. These oscillations were fairly difficult to notice in the resistive load cell data but much more obvious in the data from the fiber-optic load sensor.

#### 3.2.4. Brass Object

**Figure 15:**
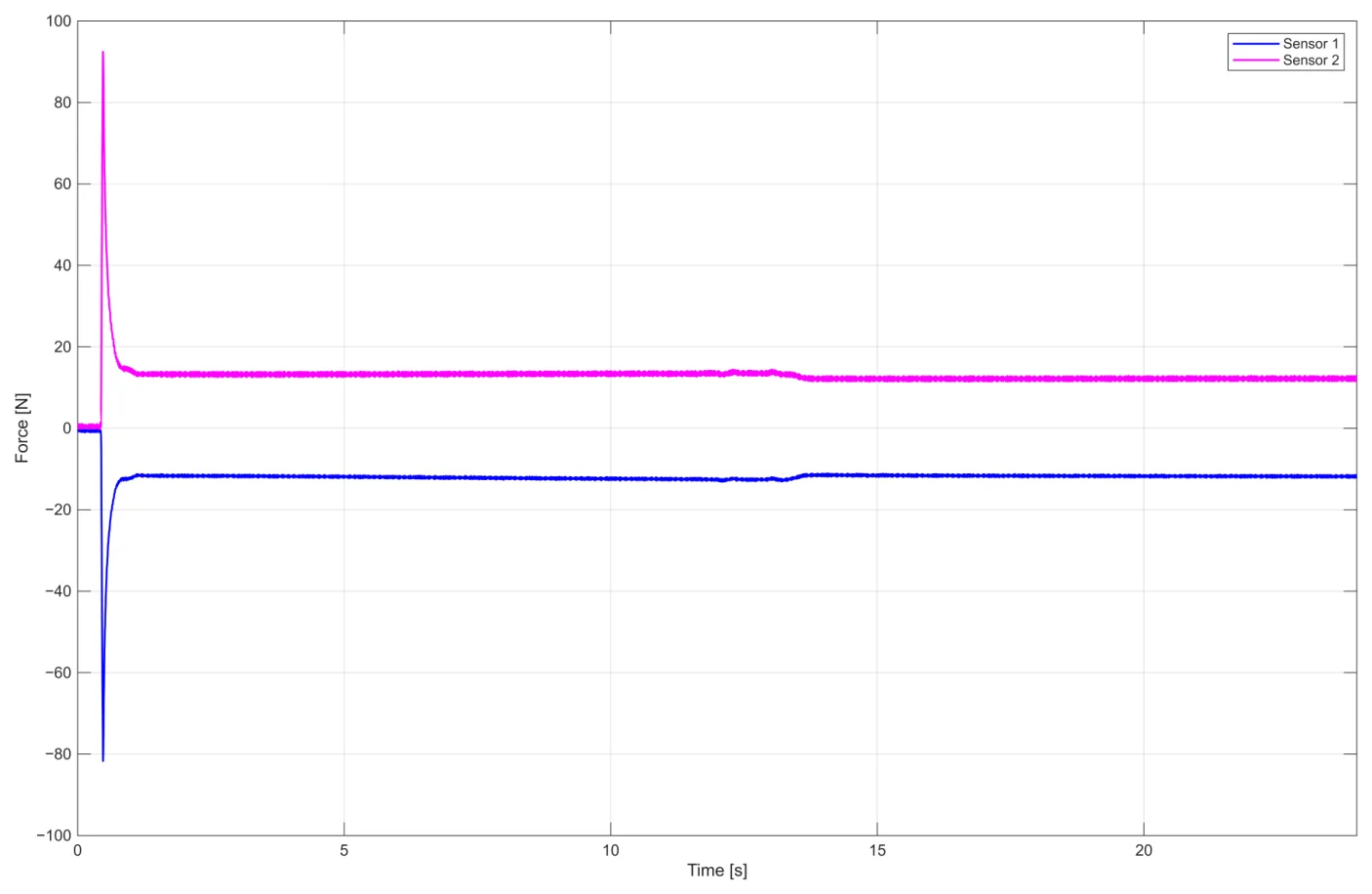
Forces measured by resistive load cells during the experiment with the brass object (Finger 1 and Finger 2).

**Figure 16:**
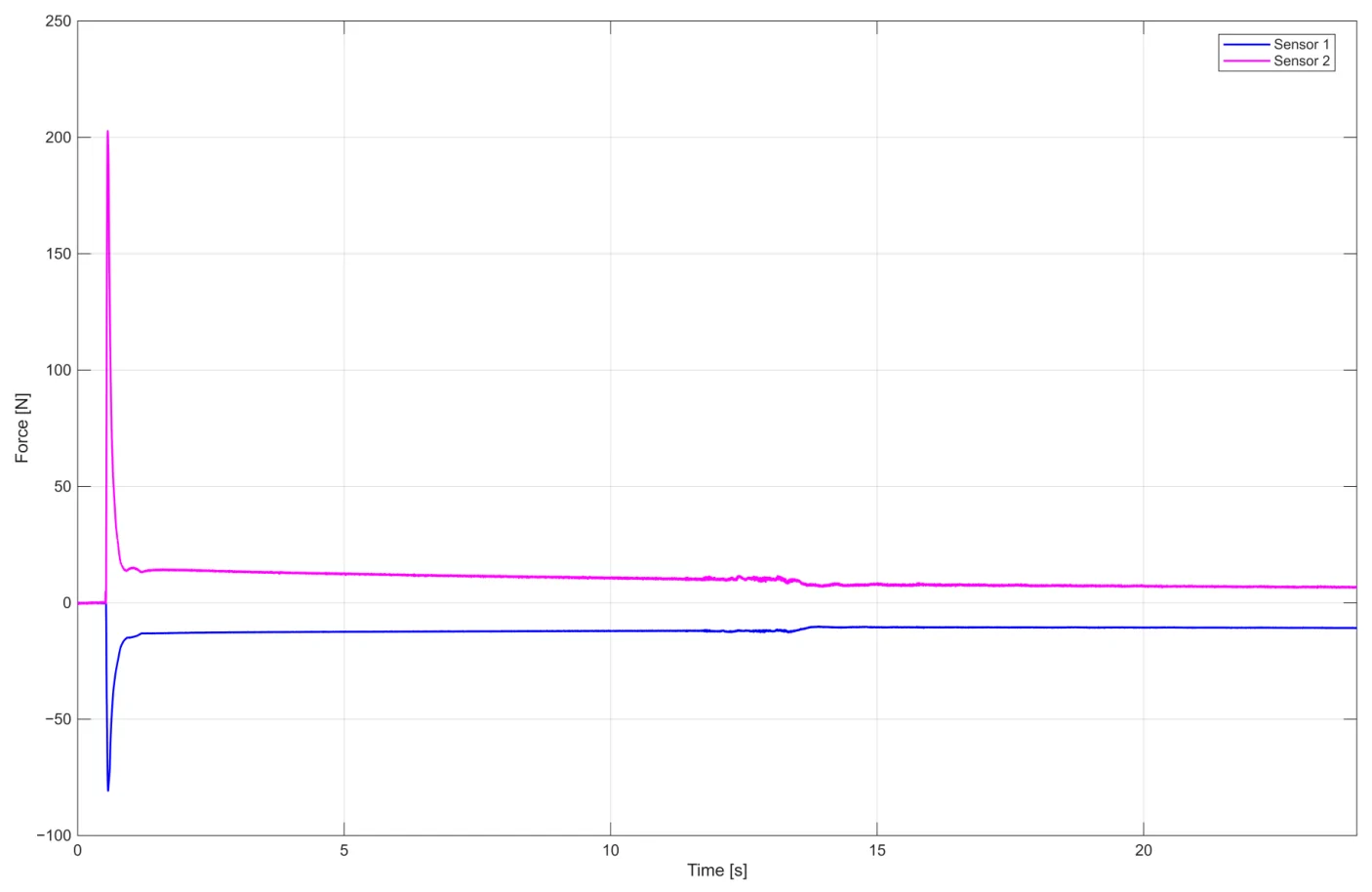
Forces measured by FBG load cells during the experiment with the brass object (Finger 1 and Finger 2).

During the experiment, noticeable slipping of the brass object was observed. This was accompanied by a sudden decrease in the grip force measured by both sensors, starting at 13 s. This is observable in Figure 15 and Figure 16. However, unlike in the experiments with the wooden cube, the change of force happened more gradually, rather than occurring as a more sudden change, as observed with the wooden object. Some oscillations slightly before the slipping (at around 12 s) were also detected. These oscillations are again more visible and cleaner in the fiber-optic load sensor data.

#### 3.2.5. Quantitative Comparison

Partitioning the idle-segment noise power shows that 86.4 ± 3.1 % of the resistive chain’s noise lies within the 45–55 Hz mains band, compared with 11.3 ± 1.3 % for the FBG chain (mean ± SD, n=5 runs; both chains band-limited to 1–95 Hz). As a spectrally flat process would place 10.6% of its power in a 10 Hz slice of that band, the resistive chain carries roughly eight times the mains-band content expected from broadband noise, whereas the FBG chain shows no measurable excess.

## 4. Discussion

### 4.1. Vibration Analysis

Comparing the sensors revealed noticeable differences in the quality of the two accelerometers. The SAA-02 fiber-optic accelerometer produced measurements with less noise and more detail. This indicates that the sensor has good sensitivity and has a substantially better signal-to-noise ratio than the MEMS accelerometer. The dominant frequencies from the SAA-02 were also more recognizable and less noisy (Figure 3). This is further supported by the signal analysis performed on the sensor data (Table 2), which showed a substantially better noise floor and signal-to-noise ratio for the FBG sensor.

With the increasing speed, an increase in the amplitude of vibrations and the range of the frequency spectrum was detected, which demonstrates a higher dynamic load and an effect of more sudden changes of velocity during robot movement. Differences in the vibrations on different axes of the effector are also observable in Figure 4 and Figure 5. A plausible cause of this could be the construction of the robot itself. At the speed of 1 m/s, new frequencies were observed, which could have been caused by resonance in the mechanical components of the robot.

Changes in vibrations caused by the weighted payload mounted on the robot were observed (Figure 6). The additional mass of the payload dampened the vibrations, mainly in the frequency range of 60 Hz to 100 Hz; however, new frequency components in the higher frequency ranges were also observed. These could have been caused by the increased load on the robot motors or other changes to the system and its resonance.

During the experiments aiming to detect improper mounting of the payload (Figure 7), the effects of a loosely mounted payload on the vibrations of the robot were clearly observable. There were significant new frequency components above 20 Hz, likely caused by the micro-movements of the payload and impacts of the payload against the flange. This could be used to detect an improper or loosened connection of the payload.

In the experiment simulating a collision, new frequency components, caused by the impact and dragging of an obstacle by the robot, were observed (Figure 8). They were caused by the mechanical interactions of the robot and the obstacle it collided with. This could allow the robot to detect a collision without other sensors present.

### 4.2. Slip Detection

In the initial experiment (Figure 9 and Figure 10), the grip force curve of the gripper holding an object during movement without any slipping was observed. A fairly constant force with some slight oscillations due to changes in the velocity of the robot was observed. The combined force measured by the sensor pairs was consistently lower than the set force of 35 N and differed slightly between sensors. This can be observed in Table 3. Plausible causes of this could be the calibration, the position of the sensor on the gripper finger and the embedding of the sensor. To help mitigate the effects of this, all comparison metrics were calculated on a per-sensor basis. There was a clear difference in clarity between the resistive and fiber-optic load sensors. The small oscillations were barely visible from the resistive load sensor data because of the noise in the signal, while the fiber-optic sensor showed them clearly. This is also further supported by the signal analysis shown in Table 3, which again shows the better noise floor and signal-to-noise ratio of the FBG sensor. Superior immunity to noise in the mains band was observed, and the majority of the resistive sensor noise was in the mains frequency band, while the FBG noise did not show any meaningful peak in this frequency band. This was consistent across all experiments. In the FBG sensor data, a slight short-term drift of the measured force was observed, while the output of the resistive load cells was more stable. This drift happened after transition events where the force changed suddenly. The measured force stabilized after a few seconds. One likely cause of this is thermal drift. The experiments were conducted in very stable laboratory conditions, with a stable ambient temperature of 23 ± 0.5 °C, which was monitored during the experiments; however, local thermal changes caused by the induced strain would explain the drift after a sudden change of grip force. Another possible cause could be the way the sensors were mounted, which could have caused slower deformation of the sensor. Even with this slight drift, the FBG sensors provided cleaner and more detailed data compared to the resistive load cells; however, if left uncompensated, this drifting could cause problems for training neural networks, since it is likely that they would learn this behavior, making them less consistent and predictable. This behavior was consistent across all experiments.

During the second experiment with the wooden cube (Figure 11 and Figure 12), slipping on both load sensors was detectable by its characteristic sudden decrease in grip force; however, some additional oscillations in the grip force in the second cycle of the trajectory were observed even when no visible slipping occurred. This could have been caused by very slight, otherwise unobservable slipping or the movement of the robot. These oscillations were also substantially clearer in the data from the fiber-optic load cell due to the much better signal-to-noise ratio, as shown in Table 3. The measured decrease in force during the slipping of the object was also more significant in the data from the FBG sensor, which is shown by the higher peak |dF/dt| that the FBG sensors recorded at slip onset, shown in Table 3. In contrast, the force lost at alarm metric shows that the FBG load sensors detected slipping after a higher loss of force compared to their resistive counterparts.

After replacing the wooden object with one made of rubber (Figure 13 and Figure 14), no significant slipping occurred, due to the high coefficient of friction of rubber. Because of this, the force curve was very consistent and only contained the slight oscillations due to the robot movement. Again, the better signal-to-noise ratio of the FBG load sensor made them considerably easier to detect compared to the resistive load cell.

During the experiments with brass (Figure 15 and Figure 16), slipping of the object was observed again. This time, the slipping was characterized by a slightly more gradual decrease in the grip force than that observed with the wooden cube. A potential cause of this is the lower coefficient of friction, which could have caused the brass object to slip more easily and gradually compared to the wooden object. The decrease in force was preceded by slight oscillations. These could have been caused by changes in velocity, as before, or by the object starting to slip. Contrary to the experiment with the wooden object, the force lost at alarm was considerably lower for the FBG sensors compared to their resistive alternatives, while the peak |dF/dt| at slip remained higher.

### 4.3. Limitations and Future Work

Several limitations should be noted: No active temperature compensation was employed, which is a likely explanation for the slight drift observed in the FBG load cell data. The noise results describe the complete measurement chains rather than the transducers alone. The advantage of the optical chain lies primarily in its immunity to mains-band interference, which improved shielding could partly offset, while the noise floor of the FBG accelerometer is set by the interrogator rather than by the sensing element. Finally, the evaluation used a single specimen for each sensor type and a limited set of grasped objects, with slip detection and vibrational analysis performed offline. The compared sensors are also in different price categories, with the FBG sensor chains being significantly more expensive compared to their alternatives. The detection of the slipping was only achieved from the force data and was not independently objectively measured. As a result, it is difficult to judge the delay between the physical slipping and the detection itself. For this reason, the force lost at alarm metric was used rather than the delay. However, the acquisition rate is not the constraint on a real-time implementation; the interrogator streams at 800 Hz with four channels active, so the dominant delay is the detection rule itself. The 50 ms differentiator window and the 20 ms persistence requirement give approximately 70 ms from slip onset to alarm. This window is imposed by the resistive chain, whose mains-band noise is not sufficiently averaged at shorter windows, whereas the optical chain clears its noise floor at 10 ms. The sampling frequency of the FBG load sensor was higher compared to its resistive alternative, which could have had an effect on the results; however, after recalculating the comparison metrics at a common 300 Hz sampling frequency, no significant changes in the results were observed. Future work will extend the comparison to more types and models of sensors, to objects with varied friction, mass, and compliance and to a real-time embedded implementation. The final goal of this research is to use the data from these sensors to train neural networks for real-time vibrational analysis and slip detection. In other future work, we intend to extend this investigation in cooperation with VÚEZ towards the use of FBG sensors on robots operating in nuclear power plant environments, where their passive all-dielectric construction, immunity to electromagnetic interference and tolerance to elevated temperature and ionizing radiation are expected to be particularly advantageous.

## 5. Conclusions

This study demonstrated that both the tested FBG sensors and their alternatives could produce data suitable for vibrational analysis and slip detection. However, the FBG sensors showed some advantages over their traditional alternatives. Across all vibration analysis experiments, fiber-optic accelerometers provided clean spectra, high sensitivity and good noise immunity. When compared to a MEMS-based MPU-6050 accelerometer, the FBG accelerometer provided cleaner and more detailed data with a lower noise floor and a better signal-to-noise ratio while also showing higher vibration amplitudes. This enabled the identification of changes in robot dynamics arising from variations in speed, payload mass, payload mounting conditions and unexpected collisions. The ability to detect such characteristic frequency components with high fidelity highlights the suitability of fiber-optic accelerometers for predictive maintenance and real-time robot health monitoring in industrial environments.

Similarly, in the slip detection experiments, the FBG load sensors in this experimental setup outperformed resistive strain gauge load cells, achieving a lower noise floor and a better signal-to-noise ratio. In some experiments, FBG load sensors also showed a faster and more significant reaction during a slip event compared to the resistive load cells. Their higher signal-to-noise ratio allowed not only the reliable identification of slip events across materials with differing friction properties, but also the detection of subtle oscillatory behavior that traditional sensors failed to capture clearly. These results indicate that FBG sensors improve the clarity, resolution, and diagnostic value of force measurements in robotic grippers, particularly during fast or dynamic manipulation.

Overall, the findings confirm that FBG accelerometers and load sensors provide robust, high-quality diagnostic data suitable for industrial vibrational analysis of robotic manipulators and other similar industrial machinery and slip detection in robotic grippers. This data may also later serve to train AI models for automatic real-time diagnostics of such machinery; however, thermal drift compensation will have to be implemented first. While both FBG sensors and their alternatives provided usable data during the experiments, the fiber-optic sensors consistently provided a lower noise floor and a higher signal-to-noise ratio, with the trade-off being higher cost and complexity, mainly because of the interrogator cost. This causes them to be less desirable in uses where the cost is the main priority and the requirements for signal clarity and accuracy are slightly lower. On the other hand, FBG sensors are very attractive in harsh environments, where the fiber-optic sensors provide clean signal, while traditional sensors are limited by noise and other environmental factors. In places where many sensors are required, the higher cost of FBG sensor chains also shrinks, since multiple sensors can use the same interrogator, which is the part that drives the cost up. As optical sensing technologies continue to mature and decrease in cost, they represent a promising direction for next-generation industrial robotics, especially in applications requiring high-precision diagnostics or operation in electromagnetically harsh environments; however, in normal conditions, their traditional alternatives provide solid performance for a lower price.

## Figures and Tables

**Table 1:** Specification of the measurement chains.

Parameter	Vibration		Grip Force	
	FBG	Reference	FBG	Reference
Sensing element	Sylex SAA-02, 3 × single-axis FBG accelerometer	MPU-6050, 3-axis capacitive MEMS	Sylex SWA-00, 2 × FBG strain sensor	2 × Wheatstone bridge load cell
Housing	Duplex 1.4462, IP67	—	SS304 (1.4301), 0.2 [mm]	Metal body
Mass (each)	280 [g]	≈10 [g]	10 [g]	—
Mounting	M5 bolt, 5 [Nm], orthogonal block	PCB, end effector	Adhesive to load cell Al profile	Gripper fingers
Measurement range	±30 [g]	±2 [g] (selected)	±1500 [με]	4 [kg] (≈40 [N])
Nominal sensitivity	90 ± 10 [pm/g]	16,384 [LSB/g]	1.2 [pm/με]	0.8 ± 0.1 [mV/V]
Calibration constant	*C* = 81.40/91.13/86.50 [pm/g] (X/Y/Z)	—	*A* = 7.75842 × 10^−7^ [$\mu \varepsilon^{-1}$]	*k* from 0.702 [kg] dead weight
Reference wavelength	1535.50/1552.58/1563.35 [nm]	—	$\lambda_{\mathrm{StrInst}}$ = 1547.5714 [nm]	—
Interrogator/DAQ	Sylex SCN-80 S-line Scan 800 + S-line Switch1x16D	Arduino Nano, USB	Sylex SCN-80 S-line Scan 800 + S-line Switch1x16D	NAU7802 24-bit ADC, TCA9548A mux, Arduino Uno R3
Wavelength range	80 [nm] (1510–1590)	—	80 [nm] (1510–1590)	—
Wavelength resolution	1 [pm]	—	1 [pm]	—
Repeatability	6 [pm] @ 25 [°C]	—	6 [pm] @ 25 [°C]	—
Accuracy (FOTR)	±13 [pm]	—	±13 [pm]	—
Sampling rate	800 [Hz] (4 channels active)	800 [Hz]	800 [Hz]	320 [Hz]
Acquisition software	Sentinel2_Dynamic v0.4.6, CSV	Python 3.12 logger, CSV	Sentinel 2.0, CSV	Arduino + Python logger, CSV
Conversion	$a=(\lambda_{\mathrm{act}}-\lambda_{\mathrm{avg}})/C$	a=N/16,384	$\varepsilon =\left(\lambda_{\mathrm{act}}-\lambda_{\mathrm{StrInst}}\right)/\left(A\;\lambda_{\mathrm{StrInst}}\right)$; $F=kEA_{c}\left(\varepsilon -\varepsilon_{0}\right)$	$F=k(R-R_{0})$

$\lambda_{\mathrm{act}}$: instantaneous Bragg wavelength; $\lambda_{\mathrm{avg}}$: trailing 5 s mean; *C*: accelerometer calibration constant; *A*: strain sensitivity coefficient; *E*, $A_{c}$: Young’s modulus and cross-sectional area of the instrumented profile; $\varepsilon_{0}$, $R_{0}$: baseline strain and unloaded bridge reading; *N*: raw 16-bit accelerometer output; *k*: force calibration factor.

**Table 2:** Quantitative comparison of the FBG (SAA-02) and MEMS (MPU-6050) accelerometer chains for the vibration test on the ABB IRB 4600 manipulator. Values are mean ± standard deviation over ten repeated runs of the experiment (n=10). Analyzed data are from the sensor comparison experiments.

Metric	FBG (SAA-02)	MEMS (MPU-6050)
Acceleration RMS [mg] ^1^	347.6±1.6	280.6±31.4
Noise floor, RMS [mg] ^1,2^	5.15±0.75	59.9±19.0
SNR [dB] ^3^	36.7±1.2	13.7±2.2
Dynamic range [dB] ^4^	52.0±1.9	38.7±1.5

^1^ 1 mg $=10^{-3}\;g_{0}$, with $g_{0}=9.81$ m/s^2^. Both chains were sampled at $f_{s}=800$ Hz and analyzed on the *X* axis. Each run comprised simultaneous FBG and MEMS acquisition; within a run, the two chains were evaluated over windows of identical length, so the runs are paired. ^2^ The noise floor is the broadband time-domain RMS of a mean-removed idle segment with the manipulator powered but stationary (mean 0.549 s, range 0.429–0.680 s); the acceleration RMS is the corresponding quantity over the active segment (mean 3.853 s, range 3.828–3.886 s). ^3^ SNR $=20\log_{10}\left(a_{\mathrm{RMS}}/n_{\mathrm{RMS}}\right)$, with both quantities being the broadband time-domain RMS values defined above. Statistics were computed on the per-run decibel values, so the tabulated mean SNR is not exactly the ratio of the tabulated mean RMS values. ^4^ Dynamic range $=20\log_{10}\left(A_{\mathrm{peak}}/n_{\mathrm{bin}}\right)$. Single-sided amplitude spectra were computed by DFT with a Hann window and coherent-gain correction (ENBW =1.5 bins) without zero padding. $A_{\mathrm{peak}}$ is the largest single-bin amplitude of the active window (Δf=0.2595 Hz on average, range 0.257–0.261 Hz), and $n_{\mathrm{bin}}$ is the median per-bin amplitude of the idle window (Δf mean 1.82 Hz, range 1.47–2.33 Hz), both evaluated over 1.0–400 Hz. Note that $n_{\mathrm{bin}}$ is a per-bin spectral level, not the broadband RMS noise floor given above, and it scales with Δf.

**Table 3:** Quantitative comparison of the FBG (SWA-00) and resistive strain gauge (NAU7802) load-sensing chains across the four slip detection experiments on the parallel gripper. Every metric is reported separately for each gripper finger. The two chains were co-located on the same finger, so columns may be compared directly. All entries are mean ± sample standard deviation over five repeated runs of each experiment. The slip metrics are reported only for the two experiments in which a slip event occurred.

Experiment	FBG (SWA-00)		Resistive (NAU7802)	
	Finger 1	Finger 2	Finger 1	Finger 2
**Noise floor, RMS [N] ^1^**				
Wood (no slip)	0.0610 ± 0.0021	0.2174 ± 0.0121	0.2236 ± 0.0040	0.4037 ± 0.0060
Wood (slip)	0.0941 ± 0.0033	0.3671 ± 0.0156	0.2108 ± 0.0047	0.3660 ± 0.0070
Rubber (no slip)	0.0592 ± 0.0044	0.1445 ± 0.0087	0.2272 ± 0.0061	0.3824 ± 0.0082
Brass (slip)	0.0557 ± 0.0032	0.2337 ± 0.0173	0.2143 ± 0.0083	0.4187 ± 0.0113
**Hold force, $|F_{\mathrm{hold}}|$ [N] ^2^**				
Wood (no slip)	12.973 ± 0.217	13.644 ± 0.534	9.928 ± 0.569	11.476 ± 0.585
Wood (slip)	12.881 ± 0.192	13.816 ± 0.628	8.857 ± 0.467	10.137 ± 0.557
Rubber (no slip)	12.084 ± 0.151	12.075 ± 0.149	9.968 ± 0.552	11.446 ± 0.568
Brass (slip)	14.522 ± 0.176	18.718 ± 0.739	12.191 ± 0.622	13.273 ± 0.808
**SNR during hold [dB] ^3^**				
Wood (no slip)	46.6 ± 0.5	36.0 ± 0.8	32.9 ± 0.5	29.1 ± 0.4
Wood (slip)	42.7 ± 0.4	31.5 ± 0.7	32.5 ± 0.6	28.8 ± 0.3
Rubber (no slip)	46.2 ± 0.5	38.5 ± 0.4	32.8 ± 0.5	29.5 ± 0.6
Brass (slip)	48.3 ± 0.4	38.1 ± 0.3	35.1 ± 0.3	30.0 ± 0.4
**Force lost at alarm [N] ^4,5^**				
Wood (slip)	1.2235 ± 0.0050	1.8698 ± 0.1752	0.6588 ± 0.0779	0.7391 ± 0.0334
Brass (slip)	0.5485 ± 0.0233	1.5037 ± 0.0718	2.6653 ± 0.1040	2.7004 ± 0.1011
**Force lost at alarm [% of $|F_{\mathrm{hold}}|$] ^6^**				
Wood (slip)	9.50 ± 0.16	13.53 ± 1.05	7.47 ± 1.14	7.30 ± 0.17
Brass (slip)	3.78 ± 0.12	8.03 ± 0.13	21.92 ± 1.52	20.39 ± 1.23
**Peak |dF/dt| at slip [N/s] ^4^**				
Wood (slip)	70.4 ± 0.5	127.4 ± 2.5	34.4 ± 3.0	36.5 ± 3.7
Brass (slip)	13.3 ± 0.2	38.5 ± 1.3	7.8 ± 0.3	12.8 ± 0.9

Rows in bold name the metric reported in the rows below them. ^1^ Estimated from the idle segment preceding the grasp in each run. ^2^ Mean force over the steady hold phase, after the grasp transient settled. ^3^ SNR $=20\log_{10}\left(\right|F_{\mathrm{hold}}\left|/n_{\mathrm{RMS}}\right)$, evaluated over the same hold phase, i.e., the ratio of the two blocks above. ^4^ Measured on the same physical slip event, so the FBG and resistive values within one run refer to an identical event. No slip occurred with the wooden object in the no-slip configuration or with the rubber object, so these metrics are undefined here. ^5^ Force lost at alarm is the deviation of the measured force from its extrapolated pre-slip level at the instant of the slip alarm, raised when |dF/dt|—from a 50 ms causal least-squares differentiator - exceeds six times its robust standard deviation during the hold phase for at least 20 ms. Being a force rather than a latency, it requires no clock alignment between the two acquisition chains. ^6^ The row above normalized by the hold force recorded by the same chain on the same finger in the same run, so that the offset between the two chains’ force scales does not bias the comparison.

## Data Availability

Data are available in a publicly accessible repository on Zenodo at https://doi.org/10.5281/zenodo.22214587.

## Abbreviations

The following abbreviations are used in this manuscript

EMI	Electromagnetic Interference
FBG	Fiber Bragg Grating
FFT	Fast Fourier Transform
MEMS	Micro-Electromechanical Systems
RMS	Root Mean Square
SNR	Signal-to-Noise Ratio
